# Minimally Invasive versus Conventional Open Surgery for Fixation of Spinal Fracture in Ankylosed Spine

**DOI:** 10.5704/MOJ.2011.005

**Published:** 2020-11

**Authors:** WH Chung, WL Ng, CK Chiu, CYW Chan, MK Kwan

**Affiliations:** Department of Orthopaedic Surgery, University of Malaya, Kuala Lumpur, Malaysia

**Keywords:** minimally invasive spinal stabilisation, long construct fixation, open surgery, spinal fracture, ankylosed spine

## Abstract

**Introduction:**

This was a retrospective study aimed to investigate the perioperative outcomes of long construct minimally invasive spinal stabilisation (MISt) using percutaneous pedicle screws (PPS) versus conventional open spinal surgery in the treatment of spinal fracture in ankylosing spondylitis (AS) and diffuse idiopathic skeletal hyperostosis (DISH).

**Material and Methods:**

Twenty-one patients with AS and DISH who were surgically treated between 2009 and 2017 were recruited. Outcomes of interest included operative time, intra-operative blood loss, complications, duration of hospital stay and fracture union rate.

**Results:**

Mean age was 69.2 ± 9.9 years. Seven patients had AS and 14 patients had DISH. 17 patients sustained AO type B3 fracture and 4 patients had type B1 fracture. Spinal trauma among these patients mostly involved thoracic spine (61.9%), followed by lumbar (28.6%) and cervical spine (9.5%). MISt using PPS was performed in 14 patients (66.7%) whereas open surgery in 7 patients (33.3%). Mean number of instrumentation level was 7.9 ± 1.6. Mean operative time in MISt and open group was 179.3 ± 42.3 minutes and 253.6 ± 98.7 minutes, respectively (p=0.028). Mean intra-operative blood loss in MISt and open group was 185.7 ± 86.4ml and 885.7 ± 338.8ml, respectively (p<0.001). Complications and union rate were comparable between both groups.

**Conclusion:**

MISt using PPS lowers the operative time and reduces intra-operative blood loss in vertebral fractures in ankylosed disorders. However, it does not reduce the perioperative complication rate due to the premorbid status of the patients. There was no significant difference in the union rate between MISt and open surgery.

## Introduction

Spinal fractures in ankylosing spondylitis (AS) patients are four times more common than the general population with lifetime incidence ranging from 5% to 15%^1-4^. Seventy percent of vertebral fractures in AS were associated with spinal cord injury at initial presentation following a trauma5. Mortality rates ranged from 7 to 32%^1,5,6^. Patients with diffuse idiopathic skeletal hyperostosis (DISH) had up to 1.5 times higher prevalence of vertebral fractures^7^. Conservative treatment plays little role in these fractures due to the long lever arm forces exerted at the fracture site. Nonsurgical treatment was associated with higher risks of complications such as pulmonary complications, thromboembolism and decubitus ulcers^8^. Therefore, many authors had described various surgical techniques such as posterior approach or combined anterior-posterior approach. However, posterior spinal stabilisation remains the most favored option^5,9,10,11^. Although combined anterior-posterior technique is advantageous from a biomechanical standpoint, it poses higher surgical risks^12^. In contrast, open surgery utilising posterior approach requires polysegmental long-construct instrumentation to counteract the forces acting at the fracture site. The role of minimally invasive stabilisation (MISt) using percutaneous pedicle screws (PPS) for vertebral fractures in ankylosed spine has not been elucidated. Previous reports on the role of MISt using PPS showed lower surgical risks i.e. reduced blood loss and operation time, but, in their report, only short segment stabilisation was investigated^13,14^. Recently, the use and advantages of long construct MISt using PPS in traumatic fractures and spinal metastasis have been investigated^15-17^. In this study, we would like to report the outcome of long construct MISt using PPS in the treatment of hyperextension spinal fractures in the ankylosed spine.

## Materials and Methods

**Fig. 1:**
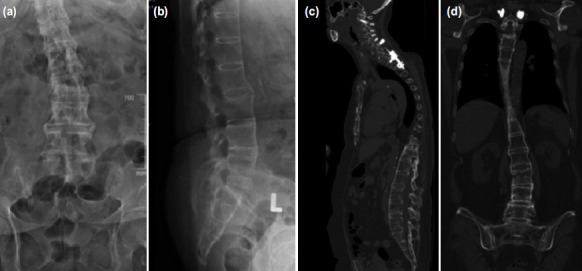
A 67-year-old lady with underlying ankylosing spondylitis presented with back pain and Frankel D neurology after a fall from standing height. Plain radiographs (a,b) and computed tomography (CT) scan (c,d) showed L3 hyperextension fracture. She has a history of C4/5 disc hyperextension fracture 2 years ago in which a long-construct posterior spinal fusion from C2 to T3 (9 instrumentation levels) was performed.

We retrospectively reviewed patients with AS and DISH who were treated for spinal fractures in a single tertiary institution from 2009 to 2017. Ethical approval was obtained. Inclusion criteria were patients who had underlying AS or DISH, who presented with vertebral fractures and treated surgically with a long construct spinal fixation (stabilisation of at least three levels above and three levels below the fractured vertebra), either by open surgery or MISt using PPS (Fig. 1). All patients underwent a computed tomography (CT) scan of the spine prior to surgery. A total of 21 patients were included.

All patients were positioned prone on a four-post frame on a Jackson table to allow good visualisation of the spinal radiographic anatomy on anteroposterior (AP) and lateral fluoroscopic views. One of the most important surgical pitfalls in AS and DISH surgery is sagittal malalignment and neurological injury during positioning and surgery. Careful, gentle positioning of a patient with an ankylosed spine is extremely important as excessive movement over the fracture site can lead to sagittal malalignment and neurological injury. In patients with hyperkyphosis, a Wilson frame might be useful to accommodate the kyphotic alignment of the spine. The height of the Wilson frame can be adjusted to allow in-situ fusion while preventing sagittal malalignment and fracture displacement which may cause neurological injury. PPS were performed simultaneously on both sides by two surgeons.

**Fig. 2:**
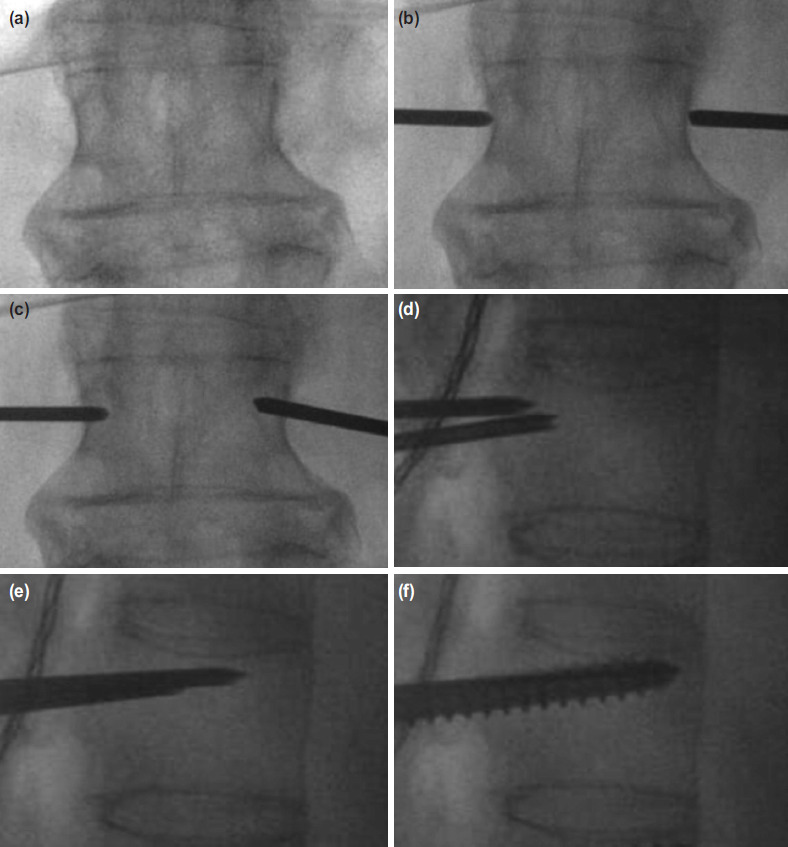
(a) Intraoperative fluoroscopic images showing steps in performing percutaneous pedicle screw insertion. A true AP view of the corresponding vertebra was taken. (b) Two 11G trocars were positioned at the lateral edge of the pedicle. (c) The trocars were advanced until the tip of the trocars approached the medial wall of the pedicles. (d) On the lateral view, the tip of the trocars should be at or slightly deeper than the posterior vertebral border. (e) The trocars were advanced until the mid-vertebral body. (f) After inserting a guide wire, the screw was inserted along the direction of the guide wire.

**Fig. 3:**
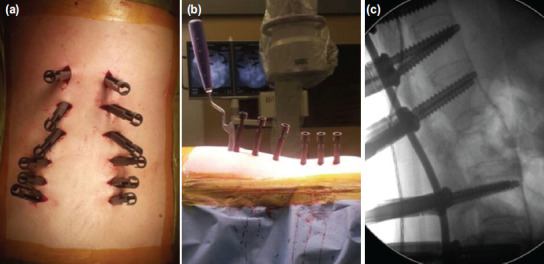
(a) Intraoperative images after insertion of all pedicle screws. (b) Rod was inserted from proximal end of the construct. (c) Fluoroscopic imaging showed in situ fixation of the fracture without any correction of preexisting deformity.

A true AP view of the corresponding vertebra was obtained, in which both superior and inferior endplates were parallel and both pedicles were equidistant from the spinous process (Fig. 2a). A skin incision of 2cm was made just lateral to the lateral edge of the pedicle for the thoracic spine and 1-2cm lateral to the lateral border of the pedicle in the lumbar spine. The fascia was incised, and the muscles were split parallel to its fibers. Two 11G trocars were positioned at the lateral edge of the pedicle (right: 3 o’clock, left: 9 o’clock) (Fig. 2b). However, different starting point were chosen for the upper thoracic level, T1-T6 vertebrae (right: 2 o’clock, left: 10 o’clock) as described by Kwan *et al*^18^. The trocar was then advanced until the tip of the trocar approached the medial wall of the pedicle on AP view (Fig. 2c). A lateral view was obtained. On the lateral view, the tip of the trocar should be at or slightly deeper than the posterior vertebral border (Fig. 2d). The trocar was then advanced until the middle of the vertebral body (Fig. 2e). A guide wire was inserted. The screw was then inserted along the direction of the guide wire, while avoiding inadvertent guide wire advancement (Fig. 2f). Once the screw position was confirmed with the lateral fluoroscopic image, the guide wire was removed. Similar steps were repeated for the rest of the planned instrumentation vertebrae (Fig. 3a).

For open surgery, the pedicle screws were inserted using ‘freehand technique’ in the thoracic and lumbar spine. In the cervical region, lateral mass screws were inserted.

Rods were contoured to allow in situ fixation of the fractured vertebra without any correction of preexisting deformity. To allow in situ fixation, we have developed an extension of the screw sleeve that would mimic the final position of the rod when seated in the screw head. The rod was then contoured with all screw sleeves and extensions positioned in a parallel alignment. At the proximal thoracic junction, rods were inserted from a caudad to cephalad direction whereas for the thoracolumbar or lumbosacral junction, rods were inserted from a cephalad to caudad direction (Fig. 3b). Nuts were inserted. Final tightening of the whole construct was performed (Fig. 3c) and lastly, deep fascia and skin were closed.

**Fig. 4:**
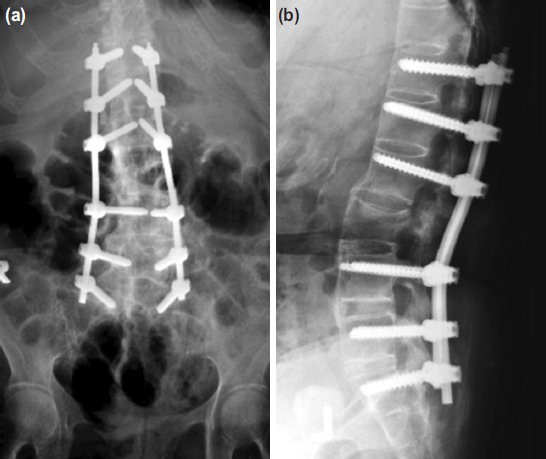
(a) Immediate postoperative AP and (b) lateral plain radiographs of the same patient treated with MISt with percutaneous pedicle screws from T12 to S1.

Post-operatively, patients with intact neurology were allowed to sit up and to ambulate to washroom with an external brace once the pain was tolerable. Continuous bladder drainage tube was removed when patients start to ambulate. Post-operative analgesia comprised of patient-controlled analgesia with morphine, oral celecoxib and acetaminophen. On day 2 or 3 post-operatively, surgical wounds were inspected. All patients were required to wear an external brace for three months. In the presence of neurological deficit, the post-operative rehabilitation protocol was decided by the spine rehabilitation team. AP and lateral standing radiographs were taken before discharge (Fig. 4).

**Fig. 5:**
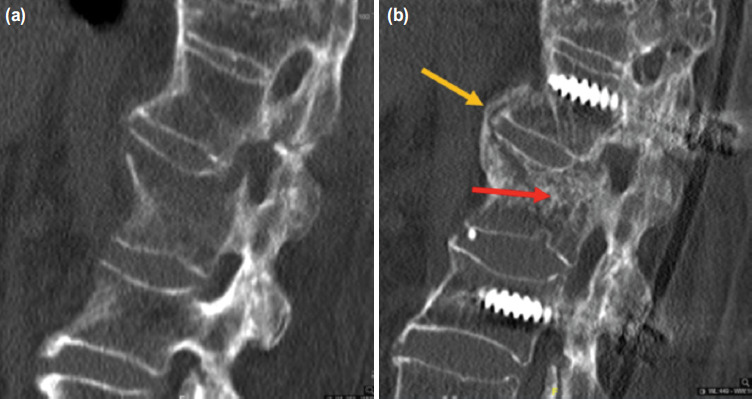
(a) CT scans showed acute hyperextension fracture evidenced by cortical breakage extending from the anterior vertebral border to the posterior vertebral border. (b) Four months postoperatively, fracture union was achieved evidenced by bridging trabeculae across the fracture site within the vertebral body (red arrow) and formation of syndesmophytes across 2 vertebral levels (yellow arrow).

All patients underwent CT scans between four to six months post-operatively. Fracture union was assessed based on the sagittal, coronal as well as axial images. A fracture union was defined as presence of bridging trabeculae across the fracture site within the vertebral body or formation of marginal or non-marginal syndesmophytes across two vertebral levels^19^ (Fig. 5).

Data collected included age, gender, diagnosis, level of injury, fracture type according to AO classification, type of surgery (open surgery or MISt), level of instrumentation, number of instrumentation levels, pre-operative and postoperative neurological function according to Frankel grade, American Society of Anesthesiologist Physical Status Classification (ASA) and Charlson comorbidity index (CCI). The perioperative outcomes that were recorded included operative time, intra-operative blood loss, complications, duration of hospital stay and union rate.

Student’s t-test was used for comparison of continuous variables while chi-squared tests were used for comparison of categorical variables between open surgery and MISt. Statistical analysis was performed using IBM SPSS Statistics for Windows, version 24.0 (IBM Corp., Armonk, N.Y., USA) with statistical significance, p value <0.05.

## Results

The mean age for this cohort was 69.2 ± 9.9 years. The mean follow-up duration was 35.3 ± 27.8 months. There were 15 males and 6 females. Seven patients were diagnosed with AS, and 14 patients with DISH. There were 17 patients with AO B3 fracture and 4 patients with B1 injury. The most common involved region was thoracic spine (61.9%), followed by lumbar (28.6%) and cervical spine (9.5%). MISt using PPS was performed in 14 patients (66.7%) whereas open surgery in seven patients (33.3%). The mean number of instrumentation level for open group and MISt group was 7.7 ± 1.7 and 7.9 ± 1.5, respectively (p=0.775). The mean ASA score was 2.3 ± 0.6 and the mean CCI score is 3.4 ± 1.4. There was no significant difference between MISt and open surgery groups in terms of age, gender, fracture type, number of instrumentation level, ASA and CCI score (Table I).

**Table I:** Demographic Data of Patients Treated with Open Surgery or MISt

	Open (n=7)	MISt (n=14)	Overall (n=21)	p value
**Age (years)**	69.3±11.5	69.1±9.5	69.2±9.9	0.976
**Gender (n(%))**				
M	6(85.7)	9(64.3)	15(71.4)	0.613
F	1(14.3)	5(35.7)	6(28.6)	
**Level of injury (n(%))**				
Cervical	2(28.6)	0(0)	2(9.5)	0.028
Thoracic	4(57.1)	9(64.3)	13(61.9)	
Lumbar	1(14.3)	5(35.7)	6(28.6)	
**Diagnosis (n(%))**				
AS	3(42.9)	4(28.6)	7(33.3)	0.638
DISH	4(57.1)	10(71.4)	14(66.7)	
**AO Classification**				
B1	2(28.6)	2(14.3)	4(19.0)	0.574
B3	5(71.4)	12(85.7)	17(81.0)	
**AO Classification (n(%))**	7.7±1.7	7.9±1.5	7.9±1.6	0.775
**ASA**	2.5±0.6	2.2±0.6	2.3±0.6	0.386
**CCI**	3.3±1.1	3.4±1.6	3.4±1.4	0.831

Abbreviations: AS = ankylosing spondylitis; DISH = diffuse idiopathic skeletal hyperostosis; ASA = American Society of Anesthesiologist Physical Status Classification; CCI = Charlson Comorbidity Index

Table II outlines the details of individual patients including their age, type of surgery, instrumentation level, preoperative and post-operative neurological status based on Frankel classification, duration of follow-up and complications.

**Table II:** Patients' Demographic and Surgical Details

No	Age (years)	Gender	Diagnosis/Fracture level	Type of Surgery	Instrumentation Level	Number of levels	Pre-op Frankel	Post-op Frankel	Follow-up (month)	Complications
1	69	M	AS/ T4	Open	T1-T7	7	E	E	99	-
2	64	M	DISH/ T11/12 disc	Open	T9-L2	6	E	E	90	-
3	50	M	AS/ C7/T1 disc	Open	C3-T6	11	A	A	66	-
4	86	M	DISH/ T10	Open	T7-L1	7	D	D	14	PUD
5	65	F	AS/ C4/5 disc	Open	C2-T3	9	C	D	34	-
6	79	M	DISH/ C6	Open	C3-T2	7	C	C	-	HAP, deceased
7	72	M	DISH/ L2	Open	T11-L5	7	E	E	45	-
8	53	M	AS/ T12	MISt	T8-L3	8	E	E	20	-
9	82	F	DISH/ L1	MISt	T7-L4	10	D	D	60	-
10	81	F	DISH/ T10	MISt	T7-L1	7	C	D	19	-
11	52	M	AS/T10/11 disc	MISt	T7-L2	8	D	D	17	Delayed union
12	70	M	DISH/ L1	MISt	T9-L4	8	D	D	15	NSTEMI
13	80	F	DISH/ L2	MISt	T9-L5	9	D	D	15	-
14	72	M	DISH/ T11	MISt	T9-L1	5	E	E	13	-
15	76	F	DISH/ T12	MISt	T7-L3	9	E	E	8	-
16	72	M	DISH/ T12	MISt	T10-L3	6	E	E	6	-
17	72	M	DISH/ T10/11 disc	MISt	T8-L3	8	D	D	35	epidural hematoma
18	60	M	DISH/T3, T7	MISt	T1-T11	11	C	C	54	-
19	67	F	AS/ L3	MISt	T12-S1	7	D	D	7	-
20	63	M	DISH/ T12	MISt	T8-L3	8	E	E	53	-
21	68	M	DISH/L1/2 disc	MISt	T10-L4	7	E	E	13	-

Abbreviations: AS = ankylosing spondylitis, DISH = Diffuse idiopathic skeletal hyperostosis, PUD = peptic ulcer disease, HAP = hospital acquired pneumonia, NSTEMI = non-ST elevation myocardial infarction

Table III illustrates the perioperative outcomes of the study. The outcomes that demonstrated significant difference were operative duration and intra-operative blood loss. The mean operative duration for MISt group was 179.3 ± 42.3 vs. 253.6 ± 98.7 minutes in the open surgery group (p=0.028). The mean intra-operative blood loss was almost five times lesser in the MISt group (185.7 ± 86.4ml) compared to the open surgery group (885.7 ± 338.8ml) (p<0.001). The mean hospital stay in the open surgery group was 42.0 ± 25.4 days compared to 21.2 ± 16.5 days in the MISt group (p=0.057).

**Table III:** Perioperative and post-operative details between open surgery and MISt

	Open (n=7)	MISt (n=14)	Overall (n=21)	p value
Operation Time (min)	253.6±98.7	179.3±42.3	204.1±73.3	0.028
Blood loss (ml)	885.7±338.8	185.7±86.4	419.1±391.9	0.000
Complication (n(%))	2(28.6)	2(14.3)	4(19.1)	0.574
Union (n(%))	6/6(100)	13/14 (92.8)	19/20 (95.0)	>0.999
Hospital stay (days)	42.0±25.4	21.2±16.5	28.1±21.7	0.057

Twelve patients (57.1%) presented with neurological deficit during admission. Among these twelve patients, eight underwent MISt and four underwent open surgery. Among patients with neurological deficit, one patient (8.3%) was classified as Frankel A, four patients (33.3%) as Frankel C and seven patients (58.3%) as Frankel D. Among those with neurological deficit, two (16.7%) showed neurological improvement. Two patients (16.7%) improved one Frankel grade. Neurological status remained the same for other patients. There was no significant difference in neurological recovery comparing MISt and the open surgery group. One patient with type B3 T10/T11 fracture, who underwent MISt T8 to L3 complained of transient neurological deficits of both lower limbs secondary to an epidural hematoma. However, the neurological deficit recovered spontaneously to Frankel D (as it was pre-operatively) prior to discharge. 85.7% of patients were ambulant (Frankel D and E) pre and post-operatively.

There were two complications in each group. There was one mortality in the open surgery group compared to none in the MISt group. The complications that were encountered included one case of upper gastrointestinal hemorrhage secondary to peptic ulcer disease (PUD), one case of hospital acquired pneumonia, one case of non-ST-elevation myocardial infarction (NSTEMI) and one case of epidural hematoma. An 86-year-old patient in the open surgery group developed upper gastrointestinal hemorrhage secondary to PUD on post-operative day 3. He remained hemodynamically stable with transfusion of three packs of allogeneic blood and medical therapy using proton pump inhibitors. A 79-year-old patient with C6 type B3 fracture with Frankel C neurological deficit underwent laminectomy C3 to C6 and instrumentation from C3 to T2, and developed hospital acquired pneumonia. Despite mechanical ventilation support post-operatively, he succumbed to death three months later. A 70-year-old patient with ischemic heart disease developed NSTEMI intra-operatively but was treated successfully with dual antiplatelet therapy.

Union rate was assessed in all patients except one in the open surgery group because of perioperative mortality. The remaining six patients (100%) in the open surgery group achieved union. In the MISt group, 13 out of 14 patients (92.8%) achieved union. One patient developed delayed union (final follow-up at 17 months). However, there was no evidence of implant loosening during the final follow-up.

## Discussion

Both AS and DISH cause similar clinical manifestations; including spinal stiffness, increased bone fragility, and difficulty with balance and gaze resulting in a higher risk of vertebral fractures^1,14^. Owing to the inherent instability of the spine resulting from ossification of surrounding soft tissues, vertebral fractures in the ankylosed spine are prone to neurological deficits^1,5,20^. Conservative treatment plays little role and is associated with poorer outcomes with spinal malalignment and persistent instability resulting in neurological impairment^21,22^.

Various surgical strategies have been described (Table IV). Few authors reported on outcomes of conventional open surgery. Sapkas *et al*^23^ performed open surgery in 20 patients; combined anterior-posterior approach in three and posterior approach in 17. All achieved union and 35% showed neurological improvement. Lu *et al*^19^ compared 14 AS patients treated with open surgery and 11 patients treated conservatively and found good results in the surgical group while 8 patients (72.7%) in conservative group had pseudoarthrosis. However, Matthews *et al*^24^ compared six AS patients treated with posterior spinal fusion and five patients treated with orthosis and reported no difference.

**Table IV:** Literature Review of Reports on Surgical Treatment of Spinal Fractures in Ankylosed Disorders

Study	n	Mean age (years)	Mean follow-up (month)	Diagnosis	Fracture level	Type of surgery	Number of instrumentation level (mean)	Operative time (min)	Blood loss (ml)	Post-operative LOS (days)	Complication	Union (%)
Sapkas *et al* (2009)	20	56.0*	60.0	AS	C, T, L	Open (PF +/- AF)	4.0	N/A	N/A	N/A	Screw loosening – 2; Infection – 1	100
Lu *et al* (2013)	22	54.2	24.0	AS	T, L	Open (PF +/- AF)	N/A	N/A	N/A	N/A	RI – 2; Empyema – 1; Infection – 1	100
Matthews *et al* (2013)	6	63.0	30.0	AS	C, T, L	Open (PF +/- AF)	N/A	N/A	N/A	N/A	Multiple	100
Kruger *et al* (2014)	10	81.5	7.9	AS & DISH	T, L	MISt	3.6	60.2 (32 – 135)	N/A	16.6 (8 22)	ROI – 1; Budd Chiari (death) – 1; RI – 2; MI – 2	N/A
Yeoh *et al* (2014)	10	68.0	22.0	AS & DISH	N/A	MISt	5.9	N/A	N/A	24.0	HAP – 1	N/A
Nayak *et al* (2015)	11	77.0	28.0	AS & DISH	T	MISt	7	227.0 (79 - 449)	251 (25 - 900)	14.4 (4 - 60)	Infection - 4	100
Moussallem *et al* (2016)	41	75.6	18.0	AS & DISH	T, L	MISt (n=25) & Open (n=16)	N/A	254.8 (MISt) 334.7 (Open)	166.8 (MISt) 1240.4 (Open)	9.6 (MISt) 16.7 (Open)	Paraplegia – 2; Revision – 4	N/A
Bredin *et al* (2017)	31	75.1	35.6	AS	T, L	MISt	5.2	N/A	N/A	5.96	Nil	100
Lindtner *et al* (2017)	20	74.7	29.2	AS	T, L	MISt (n=6) Open (n=14)	N/A	N/A	N/A	N/A	Multiple	N/A
Okada *et al* (2019)	41	77.0	N/A	DISH	N/A	MISt (n=16) & Open (n=25)	5.1 (MISt) 4.9 (Open)	168.1 (MISt) 224.6 (Open)	133.9 (MISt) 499.9 (Open)	N/A	Multiple	100
Current study	21	69.2	35.3	AS & DISH	C, T, L	MISt (n=14) & Open (n=7)	7.9	179.3 (MISt) 253.6 (Open)	185.7 (MISt) 885.7 (Open)	21.2 (MISt) 42.0 (Open)	PUD – 1; HAP (death) – 1; MI – 1; EH – 1	95.0

*median Abbreviations: n = sample size; LOS = length of stay; AS = ankylosing spondylitis; DISH = diffuse idiopathic skeletal hyperostosis; MISt = minimally invasive stabilisation; C = Cervical; T = Thoracic; L = Lumbar; PF = Posterior spinal fixation; AF = Anterior spinal fixation; N/A = not available; RI = respiratory insufficiency; ROI = removal of implant; MI = myocardial infarction; PUD = peptic ulcer disease; HAP = hospital acquired pneumonia; EH = epidural hematoma

The advent of MISt using PPS in the treatment of vertebral fractures in ankylosing disorders has reduced the morbidity associated with open surgery such as increased blood loss, higher infection rate, longer operative time, higher pain score, longer hospital stay and recovery time^13,14,25-28^. Kruger *et al*^13^ reviewed 10 patients with AS or DISH treated with MISt and reported good mid-term functional outcome with shorter operative time of 60.2 minutes (range, 32-135 min). Nayak *et al*^25^ performed MISt in 11 patients with AS or DISH and reported blood loss of 251ml, operative time of 227 minutes and good functional outcome. Yeoh *et al*^26^ evaluated 10 patients with AS or DISH and reported a mean Oswestry Disability Index (ODI) of 16 (range, 0-51), mean VAS score of 1.1 (range, 0-5) and no neurological or surgical complications. Bredin *et al*^27^ retrospectively reviewed 31 AS patients treated with MISt. All patients recovered self-sufficiency with mean Parker score of 6.73 and mean VAS score of 1.8. Three studies compared the perioperative outcomes between MISt and open group. Moussallem *et al*^14^ compared 25 patients treated with MISt and 16 patients with open surgery and documented shorter operative time (254.8 vs. 334.7 min, p=0.04), lower blood loss (166.8 vs. 1240.4ml, p<0.001), decreased transfusion rate (36.0% vs. 87.5%, p=0.001), lower complication rate (56.0% vs. 87.0%, p=0.045) and shorter hospital stay (9.6 vs. 16.7 days, p=0.008) in the MISt group. Lindtner *et al*^28^ compared six patients in MISt group and 14 patients in open group and found that the open group had higher post-operative complication rate (1.3 vs. 0.7 complications per patient). Okada *et al*^29^, in a retrospective review comparing 16 DISH patients undergoing MISt and 25 DISH patients undergoing conventional open surgery for spinal fractures, reported shorter operation duration and lower blood loss in the MISt group. Three patients in the open surgery group succumbed to death due to hypovolemic shock, respiratory failure and pneumonia, within a year of surgery.

The findings in our study were comparable to previous studies with shorter operative time, lower intra-operative blood loss and shorter hospital stay in the MISt group. No neurological deterioration was encountered. Complications and union rate were comparable to previous reports. These findings could be attributed to the utilisation of multiple “keyhole” incisions and percutaneous pedicle screws insertion which required less muscle dissection, reduced bleeding rate, reduced time for hemostasis and wound closure, increased screw placement accuracy and faster recovery. In our study, it is important to note that there was one mortality in the open surgery group compared to none in the MISt group. However, we did not find any significant difference in the perioperative complication rate between both MISt and open surgery groups. This could be probably due to a small sample size.

The number of instrumentation level was not fully investigated. Most studies only included short construct fixation (mean number of instrumentation level, Sapkas *et al*^23^: 4.0, Kruger *et al*^13^: 3.6, Yeoh *et al*^26^: 5.9 and Bredin *et al*^27^: 5.2). Only one study by Nayak *et al*^25^ documented on the usage of a long-construct fixation of 7 instrumentation levels, however, no comparison with open surgery was reported. This current study reported the usage of long-construct MISt (mean number of instrumentation level: 7.9 ± 1.6) and its perioperative benefits compared to open surgery in managing vertebral fractures in ankylosed disorders. Due to the long lever arm created by multilevel fused segments over the fracture site, a long-construct fixation with superior biomechanical stability was favored.

There were some limitations in this study. The sample size was small because the incidence of vertebral fractures in ankylosing disorders was low. Secondly, post-operative functional outcomes of patients were not reported. Further studies should be organised prospectively to evaluate the long-term functional outcomes in this group of patients. Although there were no significant differences in patients’ demographics between patients who had MISt versus open surgery, this was a non-randomised sample. Further prospective randomised study might be more useful to investigate the true differences between MISt and open surgery in treating spinal fractures among patients with ankylosed spine.

## Conclusion

MISt using PPS lowered the operative time and reduced intra-operative blood loss in fixation of vertebral fractures in AS and DISH. However, it did not reduce the perioperative complication rate because of the patients’ premorbid status. There was no significant difference in the union rate between MISt and open surgery.

## References

[1] Chaudhary SB, Hullinger H, Vives MJ. (2011). Management of acute spinal fractures in ankylosing spondylitis. ISRN Rheumatol..

[2] Mundwiler ML, Siddique K, Dym JM, Perri B, Johnson JP, Weisman MH (2008). Complications of the spine in ankylosing spondylitis with a focus on deformity correction. Neurosurg Focus..

[3] Hitchon PW, From AM, Brenton MD, Glaser JA, Torner JC (2002). Fractures of the thoracolumbar spine complicating ankylosing spondylitis. J Neurosurg..

[4] Mitra D, Elvins DM, Speden DJ, Collins AJ (2000). The prevalence of vertebral fractures in mild ankylosing spondylitis and their relationship to bone mineral density. Rheumatology (Oxford),.

[5] Westerveld LA, Verlaan JJ, Oner FC (2009). Spinal fractures in patients with ankylosing spinal disorders: a systematic review of the literature on treatment, neurological status and complications. Eur Spine J..

[6] Lukasiewicz AM, Bohl DD, Varthi AG, Basques BA, Webb ML, Samuel AM (2016). Spinal fracture in patients with ankylosing spondylitis: cohort definition, distribution of injuries, and hospital outcomes. Spine (Phila Pa 1976)..

[7] Diederichs G, Engelken F, Marshall LM, Peters K, Black DM (2011). Diffuse idiopathic skeletal hyperostosis (DISH): relation to vertebral fractures and bone density. Osteoporos Int..

[8] El Tecle NE, Abode-Iyamah KO, Hitchon PW, Dahdaleh NS (2015). Management of spinal fractures in patients with ankylosing spondylitis. Clin Neurol Neurosurg..

[9] Caron T, Bransford R, Nguyen Q, Agel J, Chapman J, Bellabarba C (2010). Spine fractures in patients with ankylosing spinal disorders. Spine (Phila Pa 1976)..

[10] Cornefjord M, Alemany M, Olerud C (2005). Posterior fixation of subaxial cervical spine fractures in patients with ankylosing spondylitis. Eur Spine J..

[11] Hunter T, Forster B, Dvorak M (1995). Ankylosed spines are prone to fracture. Can Fam Physician..

[12] He A, Xie D, Cai X, Qu B, Kong Q, Xu C (2017). One-stage surgical treatment of cervical spine fracture-dislocation in patients with ankylosing spondylitis via the combined anterior-posterior approach. Medicine (Baltimore)..

[13] Kruger A, Frink M, Oberkircher L, El-Zayat BF, Ruchholtz S, Lechler P (2014). Percutaneous dorsal instrumentation for thoracolumbar extension-distraction fractures in patients with ankylosing spinal disorders: a case series. Spine J.

[14] Moussallem CD, McCutcheon BA, Clarke MJ, Cui Q, Currier BL, Yaszemskiet MJ (2016). Perioperative complications in open versus percutaneous treatment of spinal fractures in patients with an ankylosed spine. J Clin Neurosci..

[15] Kwan MK, Lee CK, Chan CY (2016). Minimally invasive spinal stabilization using fluoroscopic-guided percutaneous screws as a form of palliative surgery in patients with spinal metastasis. Asian Spine J..

[16] Logroscino CA, Proietti L, Tamburrelli FC. Minimally invasive spine stabilisation with long implants. (2009). Eur Spine J..

[17] Roldan H, Perez-Orribo L, Spreafico M, Ginoves-Sierra M (2011). Long constructs in the thoracic and lumbar spine with a minimally invasive technique. Minim Invas Neurosur..

[18] Kwan MK, Chiu CK, Chan CYW, Zamani R, Hansen-Algenstaedt N (2017). The use of fluoroscopic guided percutaneous pedicle screws in the upper thoracic spine (T1-T6): Is it safe?. J Orthop Surg (Hong Kong)..

[19] Lu ML, Tsai TT, Lai PL, Fu TS, Niu CC, Chen LH, Chen WJ (2014). A retrospective study of treating thoracolumbar spine fractures in ankylosing spondylitis. Eur J Orthop Surg Traumatol..

[20] Lange U, Pape HC, Bastian L, Krettek C (2005). Operative management of cervical spine injuries in patients with Bechterew's disease. Unfallchirurg,.

[21] Burkus JK, Denis F (1994). Hyperextension injuries of the thoracic spine in diffuse idiopathic skeletal hyperostosis. Report of four cases. J Bone Joint Surg Am..

[22] Aoki Y, Yamagata M, Ikeda Y, Nakajima F, Nakajima A, Nakagawaet K (2013). Failure of conservative treatment for thoracic spine fracture in ankylosing spondylitis: delayed neurological deficit due to spinal epidural hematoma. Mod Rheumatol..

[23] Sapkas G, Kateros K, Papadakis SA, Galanakos S, Brilakis E, Machairas G (2009). Surgical outcome after spinal fractures in patients with ankylosing spondylitis. BMC Musculoskelet Disord..

[24] Mathews M, Bolesta MJ (2013). Treatment of spinal fractures in ankylosing spondylitis. Orthopedics..

[25] Nayak NR, Pisapia JM, Abdullah KG, Schuster JM (2015). Minimally invasive surgery for traumatic fractures in ankylosing spinal diseases. Global Spine J..

[26] Yeoh D, Moffatt T, Karmani S (2014). Good outcomes of percutaneous fixation of spinal fractures in ankylosing spinal disorders. Injury..

[27] Bredin S, Fabre-Aubrespy M, Blondel B, Falguieres J, Schuller S, Walter A (2017). Percutaneous surgery for thoraco-lumbar fractures in ankylosing spondylitis: Study of 31 patients. Orthop Traumatol Surg Res..

[28] Lindtner RA, Kammerlander C, Goetzen M, Keiler A, Malekzadeh D, Krappinger D (2017). Fracture reduction by postoperative mobilisation for the treatment of hyperextension injuries of the thoracolumbar spine in patients with ankylosing spinal disorders. Arch Orthop Trauma Surg..

[29] Okada E, Shiono Y, Nishida M, Mima Y, Funao H, Shimizu K (2019). Spinal fractures in diffuse idiopathic skeletal hyperostosis: Advantages of percutaneous pedicle screw fixation. J Orthop Surg (Hong Kong)..

